# The interaction between a leflunomide-response methylation site (cg17330251) and variant (rs705379) on response to leflunomide in patients with rheumatoid arthritis

**DOI:** 10.3389/fphar.2025.1499723

**Published:** 2025-03-20

**Authors:** Feng Zhao, Yulan Chen, Haina Liu, Lei Jin, Xin Feng, Bingbing Dai, Meng Chen, Qiao Wang, Yuxin Yao, Ruobing Liao, Junyi Zhao, Bingjia Qu, Ying Song, Lingyu Fu

**Affiliations:** ^1^ Department of Clinical Epidemiology and Evidence-based Medicine, The First Hospital, China Medical University, Shenyang, China; ^2^ Department of Rheumatology, The First Hospital, China Medical University, Shenyang, China; ^3^ Department of Rheumatology, ShengJing Hospital Affiliated of China Medical University, Shenyang, China; ^4^ Department of Rheumatology, First Affiliated Hospital of Jinzhou Medical University, Jinzhou, China; ^5^ Department of Rheumatology and Immunology, Dalian Municipal Central Hospital, Dalian, China

**Keywords:** rheumatoid arthritis, leflunomide, *PON1*, DNA methylation4, single nucleotide polymorphism

## Abstract

**Objectives:**

This research aims to reveal the mechanisms of the effect of the Paraoxonase 1 (*PON1*) gene on response to leflunomide (LEF) in rheumatoid arthritis (RA) patients, in terms of single nucleotide polymorphism (SNP), DNA methylation levels.

**Methods:**

A total of 240 RA patients enrolled were categorized into the good response group and the non-response group according to the difference in DAS28 scores between baseline and 6 months after LEF administration. The identified LEF-response cytosine-phosphate-guanines (CpGs) island (cg17330251) and its internal SNPs (rs705379, etc.) located at the *PON1* promoter were detected by Sanger sequencing and methyl target sequencing.

**Results:**

A total of 12 CpG sites at cg17330251 could be identified in our RA patients. There were significant difference between the responders and non-responders in nine CpG sites: cg17330251_2, cg17330251_3, cg17330251_4, cg17330251_6, cg17330251_7, cg17330251_8, cg17330251_9, cg17330251_10, cg17330251_12, [OR (95CI%) = 0.492 (0.250, 0.969), 0.478 (0.243, 0.940), 0.492 (0.250, 0.969), 0.461 (0.234, 0.907), 0.492 (0.250, 0.969), 0.437 (0.225, 0.849), 0.478 (0.243, 0.941), 0.421 (0.212, 0.836), 0.424 (0.213, 0.843), *P* < 0.05, respectively]. At all these nine CpG sites, the proportions of low methylation levels in the responders were higher than those in the non-responders (*P* < 0.05). In a dominant model, there was a significant difference in rs705379 wildtype CC and mutant genotypes (CT + TT) between the responders and non-responders (*P* < 0.05). The average methylation level of 12 CpG sites was lowest in rs705379-CC (median 0.229, IQR 0.195–0.287), then rs705379-CT (median 0.363, IQR 0.332–0.395), and rs705379-TT (median:0.531, IQR:0.496–0.557). The average methylation levels of 12 CpG sites were significantly negative correlated with ΔDAS28 (*r* = −0.13, *P* < 0.05). The Logistic regression indicated that combined effect of rs705379, DNA methylation of the *PON1* gene [OR (95CI%) = 1.277 [1.003, 1.626)], systemic inflammation index (SIRI) [OR (95CI%) = 1.079 (1.018, 1.143)] served as protective factors on response to LEF in RA patients.

**Conclusion:**

The RA patients with SNP-rs705379-CC, the low methylation level of *PON1*-cg17330251 and more SIRI would be susceptible of response to LEF and more suitable to choose LEF treatment.

## 1 Introduction

Rheumatoid arthritis (RA) is a persistent inflammatory autoimmune disease influencing joint synovial tissue, and the tendon sheath; it produces severe joint deformities as well as disability. RA patients also exhibit extra-articular manifestations and comorbidities, such as cardiovascular disease, respiratory disease, and so on (Figus et al., 2021). Approximately 1% of the world population has been diagnosed with RA, and this affliction is more prevalent among women than men (Smolen et al., 2016). It is currently recognized that RA patients have about a 50% increased risk of premature mortality in comparison with the general population, leading to an expected reduction in life expectancy of 3–10 years (Myasoedova et al., 2010). Sufferers of this disease often experience low quality of life and reduced life expectancy, with a simultaneous increase in usage of the healthcare system. They have a higher likelihood of experiencing unemployment, and pose a huge financial burden on individuals and society (Eriksson et al., 2015; Roodenrijs et al., 2021).

Currently, RA cannot be completely cured and research indicates that RA patients who receive timeous and effective treatment, leading to significant symptom relief, experience a reduced risk of disability and premature death (Scirè et al., 2014; Ohta and Sano, 2024). The mainstay treatment for RA involves the use of disease-modifying antirheumatic drugs, with methotrexate (MTX) and leflunomide (LEF) being first-line treatment medications among them (Abbasi et al., 2019) and LEF has similar efficacy to MTX (Qi et al., 2021), Although research indicated that LEF had a significantly lower incidence of adverse reactions in the treatment of RA compared to MTX (Wang et al., 2021), some RA patients might experience adverse reactions such as abdominal pain, diarrhea, nausea, vomiting, itching, and alopecia when taking LEF (Molina Molina et al., 2015; Koller et al., 2019). Hence, it is necessary to identify biomarkers predictive of LEF responses and ensure that patients can be administered customized therapeutic regimens that guarantee safety and efficacy.

In our previous research, LEF-related response signatures were identified by a whole-genome DNA methylation profiling using Illumina 850k methylation arrays and a targeted bisulfite sequencing assay. Following the prognostic models developed by machine learning algorithms, a seven-differentially methylated position (DMP)-based prognostic signatu7gere consisting of cg17330251, cg19814518, cg20124410, cg21109666, cg22572476, cg23403192, and cg24432675 were identified and incorporated to predict RA patients’ response to LEF following a 6-month treatment period; these were located at paraoxonase 1(*PON1*), adenosine deaminase RNA-specific B2 (*ADARB2*), ubiquitin-specific peptidase 16(*USP16*), U2AF homology motif kinase 1(*UHMK1*), and disrupted-in-schizophrenia 1 (*DISC1*), respectively (Chen et al., 2023).

Among these genes, *PON1* is an esterase enzyme that participates in maintaining the body’s oxidative balance. It is involved in the metabolism of oxidized lipids and contributes to the removal of free radicals. In 2020, J. Parada-Turska, G et al. found that the PON1 concentration was reduced in the group with high disease activity compared to the group with low disease activity (Parada-Turska et al., 2020), based on the Disease Activity Score of 28 joints (DAS28) and erythrocyte sedimentation rate (ESR) (DAS28-ESR), suggesting a potential association between *PON1* and the prognosis of RA. DNA methylation, a primary regulatory mechanism in epigenetics, is reversible through a process known as demethylation. This process can be influenced by various environmental factors including diet and medication in a time and tissue-dependent manner, so it can be used as a good therapeutic candidate. Some studies found that the activity and concentration of PON1 may be affected by both single nucleotide polymorphisms (SNPs) and DNA methylation (Zaragoza-García et al., 2021; Huen et al., 2015). K. Huen et al. showed that DNA methylation level might be negatively correlated with PON1 enzyme activity (Huen et al., 2015). In chlorpyrifos-resistant individuals with abnormal lipid profiles, the DNA methylation level of the *PON1* promoter was found to be associated with reduced expression of *PON1* mRNA (Su et al., 2019). In renal cell carcinoma, it has also been found that high methylation of *PON1* was associated with downregulation of mRNA and protein levels (Li and Yu, 2019). At 2021, research suggested that, the carriers with TT genotype at the rs705379 (−108C>T) site of *PON1* showed lower PON1 levels and enzyme activity in RA patients (Zaragoza-García et al., 2021).

Both SNP and DNA methylation not only serve as important genetic biomarkers but also play important roles in gene regulation. Thus, combining genetic and methylation data could enhance our comprehension of disease causation and prognosis. Otherwise, some studies have shown that changes in SNP sites affect the modification of DNA methylation, thereby playing a role in gene transcriptional regulation (Izzi et al., 2016; Chen et al., 2016; Hannon et al., 2018; Ardicli et al., 2024). The relationship between SNP site specific CpG and the pathogenesis of RA has been confirmed in multiple cell lines (Frank-Bertoncelj et al., 2017; Julià et al., 2017; Ai et al., 2018; Clark et al., 2020). In 2020, the research results of Alexander D et al. (Clark et al., 2020) confirmed that 37% of DNA sites associated with RA are involved in regulating changes in cis CpG methylation. Our previous identified hypomethylated cg17330251 was located at the *PON1* promoter, and interestingly, there were several functional SNPs such as the −108C/T (rs705379) locus located inside cg17330251 island, which may affect the binding of the transcription factor Sp1 (specificity protein 1, Sp1) (Deakin et al., 2003) It is worth noting that, according to the literature, there are currently no studies on the association between the *PON1* gene DNA methylation, SNP rs705379, and the response to LEF in RA patients.

To evaluate the interaction between the genetic variants and the epigenetic aberration (cg17330251) of *PON1* gene on predicting RA patient’s LEF response status. We hypothesized that allele-specific DNA methylation might affect the promoter activity of *PON1*, thereby resulting in gene expression decreasing and subsequently affecting the response of RA patients to LEF. Our study may prove helpful to the individual treatment of the RA patients.

## 2 Materials and methods

### 2.1 Study design and patient population

This study received approval from the medical ethics committee of the First Affiliated Hospital of China Medical University [approval number: (2021)89]. Patients with RA (age >18 years and of Han ethnicity) who fulfilled the 1987 revised criteria of the American College of Rheumatology (ACR) (Arnett et al., 1988) or the 2010 ACR/European League Against Rheumatism (EULAR) (Aletaha et al., 2010) criteria for the classification of RA were recruited from four Class A tertiary hospital hospitals in Liaoning Province (the First Hospital of China Medical University, Shengjing Hospital of China Medical University, the First Affiliated Hospital of Jinzhou Medical University, Dalian Municipal Central Hospital) between June 2018 to June 2020. The exclusion criteria were other autoimmune diseases (e. g. systemic lupus erythematosus, scleroderma, dermatomyositis, etc.); RA patients whose main treatment drug is not LEF but MTX or other DMARDs; pregnant or lactating patients, and patients with malignant tumors who cannot cooperate with follow-up.

All the patients were mainly treated with LEF, and could also receive combination therapy with non-steroidal anti-inflammatory drugs and small doses of corticosteroids. All participants in our study completed 6 months of follow-up. In this study, clinical data on RA patients were obtained at baseline and month six follow-up visits, as previously described, including 28-joint tender and swollen joint counts (TJC28 and SJC28), pain visual analogue scale (pain VAS), erythrocyte sedimentation rate (ESR). According to the above indicators, we were able to calculate Disease Activity Score 28-joint (DAS28) ( $DAS28=0.56\times \surd \left(TJC\right)+0.28\times \surd \left(SJC\right)+0.70\times \ln\left(ESR\right)+0.014\times VAS$ ), which assessed disease activity of RA patients. Response status for LEF treatment was determined based on changes in DAS28 (ΔDAS28 = DAS28 at baseline-DAS28 at 6 months). A good response was defined by a DAS28 improvement of >1.2 with a final DAS28 score ≤3.2, while nonresponse was defined by an improvement in DAS28 ≤ 0.6 or 0.6 ≤ DAS28 ≤ 1.2 with a DAS28 > 5.1 end-point after LEF treatment for 6 months. Patients meeting criteria falling between these categories were categorized as moderate responders (Atzeni et al., 2009; Wells et al., 2009; van Riel and Renskers, 2016). Patients exhibiting a good or moderate response were consolidated into a category termed “responder”. For the 240 patients that underwent LEF treatment for 6 months, 93 and 147 were non-responders and responders, respectively, as per EULAR criteria.

After providing standardized training to the investigators, on-site surveys were conducted in questionnaire form, covering the following: (1): Patient demographics: age, gender, etc. (2) Behavioral factors: smoking, alcohol consumption, etc. (3) Disease-related factors: number of swollen joints, number of painful joints, VAS scores, etc. Clinical indicator data, including biochemical and immunological indicators, glucose and lipid metabolism indicators, and complete blood count indicators, were collected by retrieving medical records. The data were then grouped according to the criteria established by each hospital center. We computed the neutrophil-to-lymphocyte ratio (NLR), platelet-to-lymphocyte ratio (PLR), systemic immune-inflammation index (SII), and systemic inflammation response index (SIRI) using the following formulas (Wang et al., 2023): NLR = neutrophil count/lymphocyte count; PLR = platelet count/lymphocyte count; SII = (neutrophil count × platelet count)/lymphocyte count; and SIRI = (neutrophil count × monocyte count)/lymphocyte count.

### 2.2 DNA extraction and methylation analysis

Detection of methylation levels in the promoter region of the *PON1* gene using the Targeted Bisulfite Sequencing (MethylTarget™) was conducted by the Genesky Biotechnologies Inc. (Shanghai, China). Each RA patient provided a 5 mL venous blood sample in the morning following an overnight fast. DNA was extracted from peripheral blood using the QIAamp DNA Blood Mini Kit (QIAGEN) within 72 h. Subsequently, bisulfite transformation was performed using the EZ DNA Methylation-Gold Kit (ZYMO, CA, United States). The samples were then subjected to PCR amplification with HotStart Taq polymerase (TaKaRa, Dalian, China), followed by library construction. The final step involved high-throughput sequencing on the Illumina Hiseq platform (Illumina, CA, United States) in 2 × 150 bp paired-end sequencing mode.

### 2.3 SNP selection and genotyping

Inside the cg17330251 island located at *PON1* gene promoter, there were four polymorphisms, rs705379, rs705380, rs705381, and rs553285883 in our population. SNP selection criteria include: (1): minor allele frequency (MAF) ≥10% within Asian data; (2); Hardy–Weinberg equilibrium (HWE) tests were performed on the above four SNPs: the rs705379 and rs553285883 were in HWE (*P* > 0.05), whereas the others deviated from it (*P* < 0.05) (Supplementary Table 1); (3) the transcription factor binding site prediction: on-line tools such as PROMO (https://alggen.lsi.upc.es/cgi-bin/promo_v3/promo/promoinit.cgi?dirDB=TF_8.3) and JASPAR (https://jaspar.genereg.net/) were used to predict the transcription factors potentially binding. Both PROMO website and JASPAR website suggesting that Pax-5, P53, Sp1, and ETF might be the transcription factors binding to the rs705379 site (shown in Supplementary Table 2). Transcription factor Sp1 might bind to the rs705379 locus with a score value of 16.18, and the predicted binding sequence was GGGGCGGGGG.

Genotyping analysis of SNP was performed using Sanger sequencing and then PCR primer were designed using Primer3 software, and synthesized by the Genesky Biotechnologies Inc. (Shanghai, China). Primer sequences for rs705379 were: F: GGG​TGA​GCG​CAA​TCA​GCT​TC; and R: TGG​ACT​AGG​CAC​CTA​TTC​TCT​GTC​TTC.

### 2.4 *PON1* network analysis

To elucidate the role of *PON1*, GeneMANIA (http://www.genemania.org/) was employed to discover genes exhibiting analogous functions to *PON1* and construct an interactive functional-association network (Warde-Farley et al., 2010). The network for *PON1* was established including co-expression, co-localization, physical interactions, genetic interaction and predictions to analyze function.

### 2.5 Statistical analyses

HWE was assessed using Chi-square tests. All continuous variables in this study exhibited non-normal distributions, so they were presented as medians [interquartile range (IQR)]. Categorical variables were described using frequency and percentages. Subsequent analyses included non-parametric tests and chi-squared tests as appropriate. Significant differences between groups were analyzed using the Kruskal–Wallis test. A correlation analysis was conducted by calculating the Spearman correlation coefficient (Spearman *r*). For variables with less than 10% missing data, multiple imputation methods were applied to handle missing values.

The relationship between methylation, SNP, and other variables with prognosis of RA with LEF was determined by logistic regression adjusting for age and sex. The dependent variable was RA prognosis after LEF-administering (non-responder was assigned as 0). *PON1* methylation levels were divided into three groups (low, medium, high) according to quartiles, the cut-offs were 25th percentile and 75th percentile. Methylation levels at cg17330251 (significant sites) were assigned values of 0 for hypermethylation, 1 for intermediate methylation, and 2 for hypomethylation. The TT, CT, and CC of the *PON1* rs705379 locus were assigned scores of 0, 1, or 2. The assigned values were then summed up to derive the composite genetic protective score (Score), which was further categorized into quartiles for analyzing its association with the prognosis of RA response to LEF. The score was incorporated into the logistic regression analysis to estimate the impact of multiple omics loci in the *PON1* gene on predicting the response of RA patients to LEF.

Statistical analyses were performed using IBM SPSS Statistics for Windows, Version 25.0 (Released 2017; IBM Corp., Armonk, New York, United States) and R software (Version 4.3.0, Vienna, Austria) along with the RStudio interface (Version 2023.03.0, Boston, MA, United States). GraphPad Prism 9.0.0 was used to plot the results. The significance level was set at *P* < 0.05.

## 3 Results

### 3.1 Demographic characteristics of study participants

A total of 240 patients with RA were enrolled in this study who could be divided into the response group (*n* = 147) and the non-response group (*n* = 93). Descriptive analysis was performed on the basic information of the patients, and the results are summarized in Supplementary Table 3. Monocyte (MONO) (*P* = 0.020) showed a statistically significant difference between two groups. After univariate Logistic analysis, the plateletcrit (PCT) (OR = 0.608 [0.309, 1.197)], MONO [OR = 1.900 (1.101, 3.278)], platelet count (PLT) [OR = 0.642 (0.328, 1.256)], and systemic inflammation response index (SIRI) [OR = 1.084 (1.022, 1.149)] were potential risk factors for RA prognosis according to the criterion of *P* < 0.2.

### 3.2 The relationship between the DNA methylation level of *PON1*-cg17330251 and prognosis of RA response to LEF

After detection of methylation levels, there were a total of 12 CpG sites at *PON1-*cg17330251 in our patients. There were significant difference between the responders and non-responders in 9 CpG sites, cg17330251_2, cg17330251_3, cg17330251_4, cg17330251_6, cg17330251_7, cg17330251_8, cg17330251_9, cg17330251_10, cg17330251_12, (OR (95CI%) = 0.492 (0.250, 0.969), 0.478 (0.243, 0.940), 0.492 (0.250, 0.969), 0.461 (0.234, 0.907), 0.492 (0.250, 0.969), 0.437 (0.225, 0.849), 0.478 (0.243, 0.941), 0.421 (0.212, 0.836), and 0.424 (0.213, 0.843), respectively). At all these nine CpG sites, the proportions of low methylation levels in the responders were higher than those in the non-responders (*P* < 0.05). RA patients with low methylation levels would be a significantly better prognosis than those with moderate methylation levels. Specifically, at the cg17330251_6, RA patients with low methylation levels would be also have a better prognosis than those with high methylation levels [OR (95CI%) = 0.478 (0.243, 0.940)] (Supplementary Table 4).

### 3.3 The association between *PON1*-rs705379 and prognosis of RA response to LEF

In dominant model, there was significant difference in rs705379 wildtype CC and mutant genotypes (CT + TT) between the responders and non-responders (*P* < 0.05). More CC-carriers were found in the response group (34.01%) than in the non-response group (21.50%) [OR (95%CI) = 0.532 (0.291, 0.973)]. In codominant and recessive models, there were no significant differences in the distribution of genotypes at the rs705379 locus of the *PON1* gene in responders and non-responders (*P* > 0.05). The results are shown in Table 1.

**TABLE 1:** Genotype frequencies and risk estimates of *PON1* rs705379 in the RA responders and non-responders.

Model type	Genotype	Responders (n, %)	Non-responders (n, %)	χ^2^	*P* ^a^	OR (95%CI)	*P* ^b^
Codominant	CC	50 (34.01)	20 (21.50)	4.404	0.111	Ref	
	CT	63 (42.86)	49 (52.69)			0.513 (0.270,0.973)	0.041
	TT	34 (23.13)	24 (25.81)			0.572 (0.272,1.202)	0.141
Dominant	CC	50 (34.01)	20 (21.50)	4.314	0.038	Ref	
	CT + TT	97 (65.99)	73 (78.50)			0.532 (0.291,0.973)	0.041
Recessive	CC + CT	113 (76.87)	69 (74.19)	0.223	0.637	Ref	
	TT	34 (23.13)	24 (25.81)			0.879 (0.479,1.611)	0.676
Allele	C	163 (55.4)	89 (47.8)	2.643	0.105	Ref	
	T	131 (44.6)	97 (52.2)			0.737 (0.510,1.066)	0.105

^a^ Chi-square test.

^b^ Logistic regression adjusted for age, sex; OR, odds ratio; CI, confidence interval; Data are presented as *n* (%). Bold values indicate the positive locus determined by statistical analysis.

### 3.4 The association among rs705379, average methylation levels of cg17330251 of *PON1* and ΔDAS28

**FIGURE 1:**
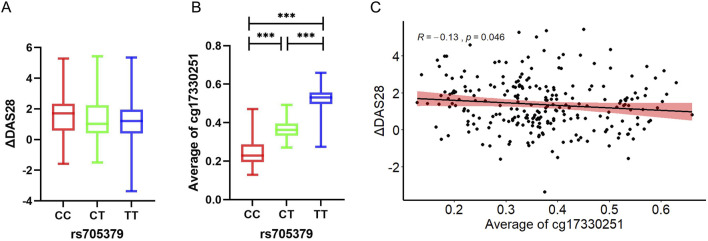
Correlations among rs705379, average methylation levels of cg17330251 of *PON1* and ΔDAS28. ***P < 0.001. **(A)** ΔDAS28 in each genotype at rs705379; **(B)** Average methylation level of cg17330251 in each genotype at rs705379; **(C)** Correlation between average methylation level of cg17330251 and ΔDAS28.

There were significant statistical differences observed in ΔDAS28 among different genotypes at the rs705379 (Figure 1A). Notably, there were significant differences in average methylation levels of 12 CpG sites between the genotype CC, genotype CT and genotype TT at the rs705379 (*P* < 0.001, respectively) (Figure 1B). The average methylation level was lowest in rs705379-CC (median 0.229, IQR 0.195–0.287), then rs705379-CT (median 0.363, IQR 0.332–0.395), and rs705379-TT (median:0.531, IQR:0.496–0.557). The results of the correlation analysis indicated that the average methylation levels of 12 CpG sites were significantly negatively correlated with ΔDAS28 (*r* = −0.13, *P* < 0.05), as shown in Figure 1C.

### 3.5 Multifactor analysis for the prognosis of LEF-taking RA patients

Two models were constructed by the Logistic regression (Table 2). In Model 1, the genetic protective score was analyzed by univariate logistic analysis and there was a significant association with the prognosis of RA after LEF administration. In Model 2, the genetic protective score and four potential risk factors for RA prognosis (PCT, MONO, PLT and SIRI) were analyzed by logistic regression, and the result showed that the genetic protective score and SIRI were significantly associated with the RA prognosis. In both the models, the increased protective score was associated with better response to LEF in RA patients (OR = 1.313, 95%CI = 1.035–1.665, *P* = 0.025; OR = 1.277, 95%CI = 1.003–1.626, *P* = 0.048). More SIRI was also related to better response to LEF in RA patients (OR = 1.079, 95%CI = 1.018–1.143, *P* = 0.011). In Model 2, there was no issue of collinearity among the variables (*P* > 0.05, Supplementary Table 5).

**TABLE 2:** The results of multifactor Logistic regression analysis combining patient genetic factors with traditional prognostic factors.

Model	Indicators	β	S.E.	Wald	df	OR (95%CI)	*P*
Model 1							
	Genetic score	0.272	0.121	5.046	1	1.313 (1.035,1.665)	0.025
	Constant	−0.184	0.311	0.351	1	0.554	0.832
Model 2							
	Genetic score	0.244	0.123	3.927	1	1.277 (1.003,1.626)	0.048
	SIRI	0.076	0.030	6.501	1	1.079 (1.018,1.143)	0.011
	Constant	−0.380	0.323	1.378	1	0.684	0.240

β, regression coefficient; S.E., standard error; df, degrees of freedom; OR, ratio of ratios; CI, confidence interval; Bold values indicate the positive locus determined by statistical analysis.

### 3.6 *PON1* network analysis

**FIGURE 2:**
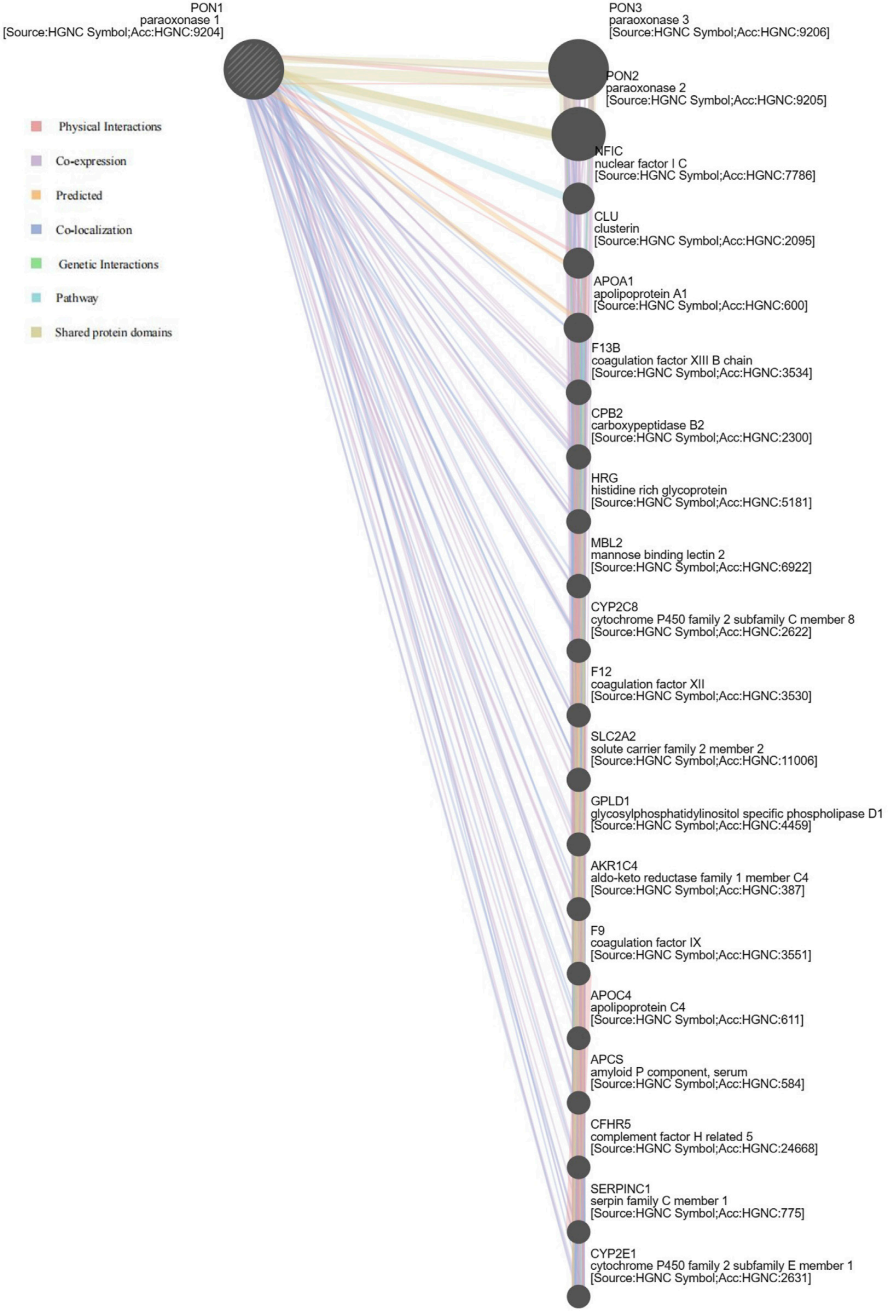
Biological interaction network of *PON1*. Different colors represent diverse bioinformatics methods.

A gene–gene interaction network for *PON1* was constructed, and its function was analyzed using the GeneMANIA database (Figure 2). Functional analysis indicated that 20 proteins were correlated with PON1, including PON3, PON2, NFIC, CLU, and APOA1. These proteins showed the greatest correlation in terms of the icosanoid metabolic process (False discovery rate = 1.986 × 10^−5^), complement activation, lipoprotein particle, plasma lipoprotein particle, protein-lipid complex (False Discovery Rate = 3.937 × 10^−5^). Additionally, these proteins were correlated in terms of fatty acid derivative metabolic process and fatty acid metabolic process. Importantly, *PON1* gene exhibits co-expression and co-localization relationships with members of the cytochrome P450 family, such as *CYP2C8* and *CYP2E1*, as well as *GPLD1* gene, which were associated with the drug metabolic processes and drug responses (Supplementary Table 6).

## 4 Discussion

In this study, the interactions between genetics, DNA methylation at *PON1* susceptibility cg17330251 and functional SNP rs705379 were analyzed. We initially discovered that RA patients with low methylation levels would be more responsive to LEF than those with medium methylation levels. After that, RA patients with the *PON1*-rs705379-CC genotype would be susceptible to response to LEF and better suited to adoption of LEF treatment. We then found that a reduction of methylation levels led to a significant increase in ΔDAS28. More importantly, in the carriers of rs705379-CC, the methylation levels of the cg17330251 significantly decreased, and then ΔDAS28 would significantly increase. All these suggested that functional rs705379 site can alter CpG site modification and thus influence epigenetic regulation as associated with prognostic response to LEF in RA patients. Our results may represent a functional link between genetic variation and an epigenetic modification for *PON1* expression, and further effects on patient susceptibility to LEF. To the best of our knowledge, this is the first study to explore the mechanism of the *PON1* gene on RA patients’ response to LEF based on the integration of genetic and epigenetic factors.

**FIGURE 3:**
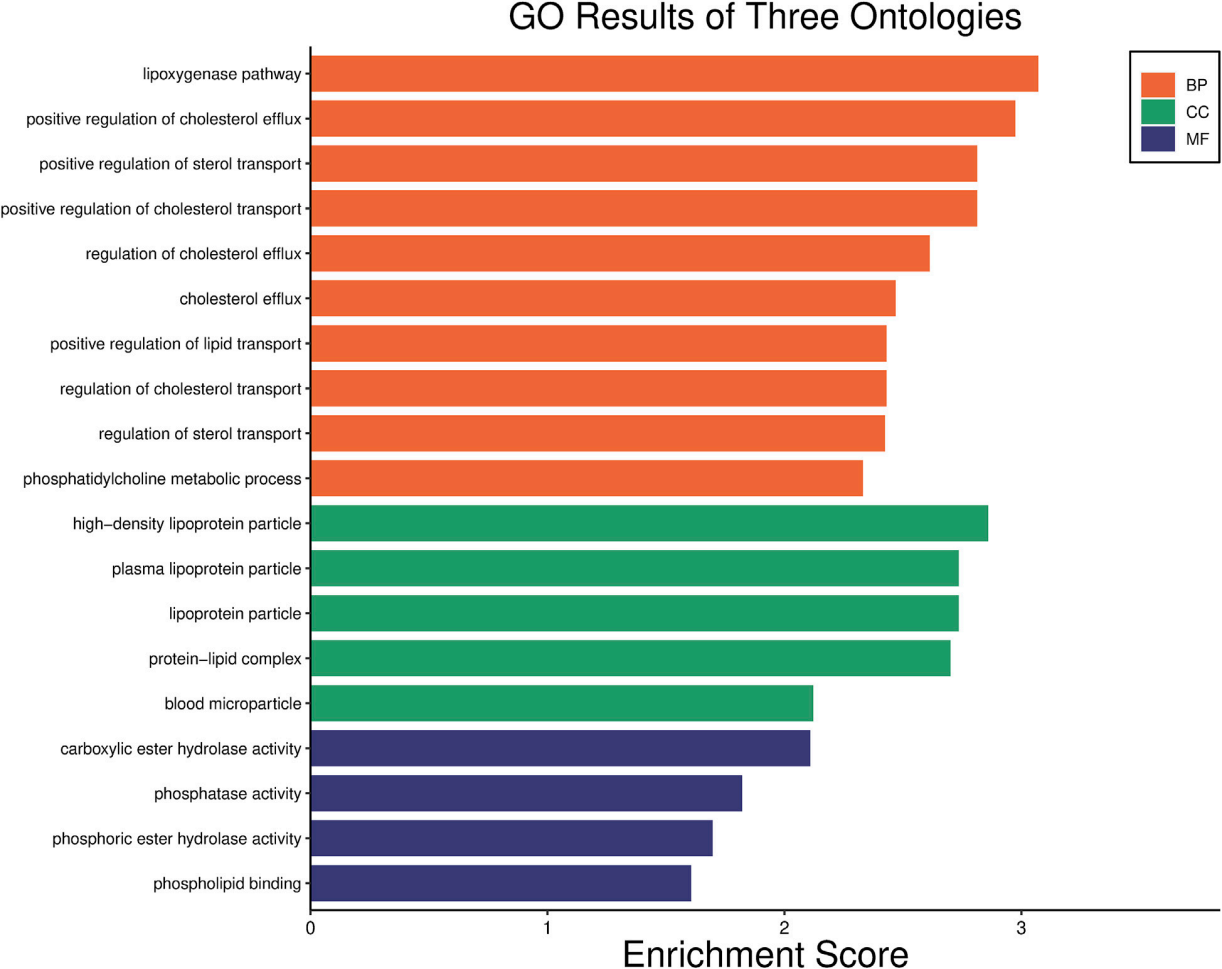
GO enrichment analysis of *PON1* gene.

Our previous research revealed that *PON1*, *ADARB2*, *USP16*, *UHMK1*, and *DISC1* might serve as biomarkers for predicting the response to LEF in RA patients (Chen et al., 2023). Through Gene Ontology (GO) bioinformatics enrichment analysis, we found that the *PON1* gene was primarily involved in the ester oxygenase pathway (Figure 3). Its expressed product, PON1, is an esterase that metabolizes oxidized lipids and organophosphates. It participates in eliminating free radicals to maintain oxidative balance, protecting both high-density lipoprotein (HDL) and low-density lipoprotein (LDL) from oxidation, and playing a crucial anti-inflammatory role (Aviram et al., 1998). Research on the *PON1* gene has mainly focused on cardiovascular diseases, with relatively limited studies in RA at present. A study from 2020 proposed that the overexpression of the human *PON1* transgene was linked to a decrease in inflammatory arthritis. This effect may be closely related to increased circulating PON1 activity, the upregulation of the hepatic glutathione pathway, and a decrease in circulating biologically active mediators. These findings suggested that targeting *PON1* can potentially be a therapeutic strategy for joint diseases, including RA (Charles-Schoeman et al., 2020). The findings of this study align with the aforementioned perspectives.

The current study implied that the hypermethylation pattern within the promoter region of the *PON1* gene might be a contributing factor to the insufficient response to LEF in RA. Methylation of CpG islands situated in the gene’s promoter region is a consistently acknowledged mechanism for expression silencing (Vanderkraats et al., 2013). The inadequate response to LEF in RA might be attributed to elevated methylation levels influencing aromatase activity (a metric indicative of the amount of PON1 protein in the bloodstream) (Huen et al., 2015; Diels et al., 2021; de la Iglesia et al., 2014). SNP can affect the methylation of nearby CpG sites, leading to differential methylation levels between different genotypes, a phenomenon known as allele-specific methylation (ASM) (Schalkwyk et al., 2010; Shoemaker et al., 2010). The methylation levels of various CpG sites within the cg17330251 methylation island differed significantly among genotypes at the rs705379, providing robust evidence for the occurrence of ASM, which is consistent with the findings reported by Huen et al. (2015). More particularly, Diels et al. (2021) found that TT-genotype carriers of the regulatory variant rs705379 had higher DNA-methylation values at the *PON1* promoter region and such a relationship was found in our study. 22% of ASM genes also exhibited allele-specific gene expression, and this expression was not tissue-specific (Gertz et al., 2011). N. Gupta et al. demonstrated that individuals with genotypes CT and TT exhibited a lower PON1 activity compared to those with genotype CC (Gupta et al., 2011). Although we had performed mRNA expression analysis of the PON1 gene in our blood samples, unfortunately, due to the low expression of the *PON1* gene in these samples, the mRNA expression was only detected in a few samples and could not be compared between the responders and non-responders. M. J. Bonder et al. found a negative correlation between DNA methylation levels in the *PON1* promoter region and its mRNA expression levels in liver samples (Bonder et al., 2014). When verifying the mRNA expression level of this gene, liver samples need to be collected, which is very difficult in RA patients.

Recently, a number of studies proved that genetic and epigenetic factors play a crucial role as prognostic biomarkers in RA (Sánchez-Maldonado et al., 2021; Manuel Sánchez-Maldonado et al., 2020; Gravand et al., 2023; Ravaei et al., 2022). DNA methylation, as the most studied epigenetic modification, is thought to play an important role in RA disease pathogenesis and in mediating the relationship between genetic variants and patient outcomes (Nair et al., 2017; Srivastava and Rasool, 2024; Nair et al., 2020). The regulatory mechanisms of gene expression are intricate and remain not completely elucidated. we hypothesized that the presence of the C nucleotide at the upstream C/T polymorphic site could lead to low methylation of the adjacent C. Its mechanism may be that rs705379-C had a stronger binding affinity with the transcription factor Sp1, which initiated transcription, forming a DNA-RNA hybrid. This can prevent DNA methyltransferase from binding to the promoter region, resulting in hypomethylation (Ginno et al., 2012; Höller et al., 1988; Chen and Zhang, 2020). Delightfully, Deakin and others have already verified, through a dual-luciferase assay, rs705379 was located in the binding sequence of the transcription factor Sp1 and the C allele exhibited a stronger binding ability to the transcription factor Sp1 than the T allele (Deakin et al., 2003). The results of the data analysis in this study could confirm this mechanism, and we did not consider it necessary to perform dual luciferase experiments to verify it again.

A comprehensive analysis of genetic factors and traditional clinical prognostic indicators prompted that higher genetic protective score (SNP and DNA methylation) and SIRI were related to the better response to LEF in RA patients. This indicates that there is significant potential for clinical application in assisting physicians with the development of treatment plans for patients with RA. By utilizing these insights, healthcare providers can create more tailored and effective strategies to manage the disease. Ultimately, this could enhance patient outcomes and improve the overall management of RA. Considering that the study population was limited to four hospitals in Liaoning Province, China, the generalizability of the results obtained in this study may be limited. Further validation in a larger and more diverse population is necessary to enhance the robustness and applicability of the findings. Besides, in this study, multiple testing corrections for CpG site analysis (FDR or Bonferroni adjustment) was not applied, due to the subtlety of the CpG site effects and the aim is to broadly screen for methylation sites that may be associated with prognosis in the initial exploratory stage. This could introduce false-positive outcomes. Therefore, we intend to validate the results in an expanded population sample.

## 5 Conclusion

In conclusion, to the best of our knowledge, this study represents the first investigation into the prognostic implications of the rs705379 of *PON1*, DNA methylation levels in patients with RA undergoing LEF treatment. The conserved genotypes of polymorphisms in *PON1*, hypomethylation of the promoter region collectively exert a synergistic impact on the prognosis of RA. The present study offers a novel perspective on the role of *PON1* in the prognosis of RA.

## Data Availability

The raw data supporting the conclusions of this article will be made available by the authors, without undue reservation.

## Glossary

ADARB2: Adenosine deaminase RNA-specific B2
Anti-CCP: Anti-cyclic citrullinated peptide
ASM: Allele-specific methylation
CpG: Cytosine-phosphate-guanine
CRP: Reactive protein
DISC1: Disrupted-in-schizophrenia 1
DAS28: Disease Activity Score of 28 joints
EULAR: European League Against Rheumatism
ESR: Erythrocyte sedimentation rate
GLU: Glucose
GO: Gene ontology
HDL: High density lipoprotein
IgA: Immunoglobulin A
IgG: Immunoglobulin G
IgM: Immunoglobulin M
LDL: Low density lipoprotein
LEF: Leflunomide
LY: Lymphocyte
MONO: Monocyte
NLR: Neutrophil-to-lymphocyte ratio
MTX: Methotrexate
OR: Odds ratio
PCR: Polymerase chain reaction
PCT: Plateletocrit
PLR: Platelet-to-lymphocyte ratio
PLT: Platelet count
PON1: Paraoxonase 1
RA: Rheumatoid arthritis
RBC: Red blood cell
RF: Rheumatoid arthritis factor
SII: Systemic immune-inflammation index
SIRI: Systemic inflammation response index
SNP: Single nucleotide polymorphism
Sp1: Specificity protein 1
TC: Serum total cholesterol
TG: Triglyceride
TOL: Tolerance
UHMK1: U2AF homology motif kinase 1
USP16: Ubiquitin-specific peptidase 16
VAS: Visual analogue scale
VIF: Variance inflation factor
95%CI: 95%Confidence interval
TJC28: 28-joint tender joint counts
SJC28: 28-joint swollen r joint counts
