# Supplementary material for: The interaction between a leflunomide-response methylation site (cg17330251) and variant (rs705379) on response to leflunomide in patients with rheumatoid arthritis
Source: Front Pharmacol. 2025 Mar 20;16:1499723. doi: 10.3389/fphar.2025.1499723 (PMC11965123; doi:10.3389/fphar.2025.1499723)
Supplement: Supplementary file 1 [file Table1.docx]

Supplementary Material

The interaction between a leflunomide-response methylation site (cg17330251) and variant (rs705379) on response to leflunomide in patients with rheumatoid arthritis

Feng Zhao*, Yulan Chen, Haina Liu, Lei Jin, Xin Feng, Bingbing Dai, Meng Chen, Qiao Wang, Yuxin Yao, Ruobing Liao, Junyi Zhao, Bingjia Qu, Ying Song

*** Correspondence:** Lingyu Fu: [fulingyucmu@sina.com](mailto:fulingyucmu@sina.com).

# Supplementary Figures and Tables

## Supplementary Tables

**Supplementary Table 1. Results of Hardy-Weinberg equilibrium test for SNPs of the *PON1* gene.**

| Genotypes | Actual frequency | Theoretical frequency | HWEχ^2^ | *P* |
| --- | --- | --- | --- | --- |
| *PON1* rs705379 |  |  |  |  |
| CC | 70 | 66 | 1.072 | 0.589 |
| CT | 112 | 120 |  |  |
| TT | 58 | 54 |  |  |
| *PON1* rs705380 |  |  |  |  |
| GG | 24 | 66 | 132.605 | ＜0.01 |
| GC | 195 | 110 |  |  |
| CC | 3 | 46 |  |  |
| *PON1* rs705381 |  |  |  |  |
| TT | 8 | 57 | 160.760 | ＜0.01 |
| TC | 216 | 118 |  |  |
| CC | 13 | 62 |  |  |
| *PON1* rs553285883 |  |  |  |  |
| GG | 238 | 238 | 0.004 | 0.997 |
| GA | 2 | 2 |  |  |
| AA | 0 | 0 |  |  |

**Supplementary Table 2. Transcription factor binding prediction results for the rs705379 of the *PON1* gene from the PROMO website.**

| ID | Transcription factor | Dissimilarity （%） | Random Expectation | Sequence |
| --- | --- | --- | --- | --- |
| T00070 | Pax-5 | 1.54% | 0.07288 | GGGCGGG |
| T00671 | P53 | 3.38% | 0.07288 | GGGCGGG |
| T00759 | Sp1 | 0.00% | 0.00019 | GGGGCGGGGC |
| T00270 | ETF | 7.87% | 0.00712 | GCGGGGCGGGC |

**Supplementary Table 3. Demographic characteristics in responders and non-responders.**

|  | Responders  （n=147） | Non-responders  （n=93） | Statistics  （χ^2^/t） | *P* | OR (95%CI) | *P*^*^ |
| --- | --- | --- | --- | --- | --- | --- |
| Age M (P25, P75) | 58(51,66) | 59(51,66) | -0.46^a^ | 0.645 | 0.996(0.976,1.017) | 0.709 |
| Gender n(%) |  |  | 0.283^b^ | 0.595 |  |  |
| men | 32(21.77) | 23(24.73) |  |  | Ref | |
| women | 115(78.23) | 70(75.27) |  |  | 1.181(0.640,2.179) | 0.595 |
| Hypertension n(%) |  |  | 0.481^b^ | 0.488 |  |  |
| no | 128(87.07) | 78(83.87) |  |  | Ref | |
| yes | 19(12.93) | 15(16.13) |  |  | 0.772(0.371,1.607) | 0.489 |
| Diabetes n(%) |  |  | 0.987^b^ | 0.320 |  |  |
| no | 134(91.96) | 88(94.62) |  |  | Ref | |
| yes | 13(8.84) | 5(5.38) |  |  | 1.707(0.588,4.957) | 0.325 |
| Smoke n(%) |  |  | 1.152^b^ | 0.283 |  |  |
| no | 129(87.76) | 77(82.80) |  |  | Ref | |
| yes | 18(12.24) | 16(17.20) |  |  | 0.672(0.324,1.394) | 0.285 |
| Alcohol n(%) |  |  | 1.344^b^ | 0.246 |  |  |
| no | 131(89.12) | 87(93.55) |  |  | Ref | |
| yes | 16(10.88) | 6(6.45) |  |  | 1.771(0.667,4.703) | 0.251 |
| ESR n(%) |  |  | 0.811^b^ | 0.368 |  |  |
| low | 35(23.81) | 27(29.03) |  |  | Ref | |
| high | 112(76.19) | 66(70.97) |  |  | 1.309(0.728,2.354) | 0.368 |
| CRP n(%) |  |  | 0.123^b^ | 0.726 |  |  |
| low | 35(23.81) | 24(25.81) |  |  | Ref | |
| high | 112(76.19) | 69(74.19) |  |  | 1.113(0.611,2.028) | 0.726 |
| RF n(%) |  |  | 0.031^b^ | 0.860 |  |  |
| - | 38(25.85) | 25(26.88) |  |  | Ref | |
| + | 109(74.15) | 68(73.12) |  |  | 1.055(0.585,1.900) | 0.860 |
| Anti-CCP n(%) |  |  | 0.886^b^ | 0.352 |  |  |
| - | 26(17.69) | 21(22.58) |  |  | Ref | |
| + | 121(82.31) | 72(77.42) |  |  | 1.357(0.712,2.587) | 0.353 |
| IgA n(%) |  |  | 0.546^b^ | 0.460 |  |  |
| low | 116(78.91) | 77(82.8) |  |  | Ref | |
| high | 31(21.09) | 16(17.2) |  |  | 1.286(0.659,2.510) | 0.461 |
| IgG n(%) |  |  | 1.212^b^ | 0.271 |  |  |
| low | 113(76.87) | 77(82.80) |  |  | Ref | |
| high | 34(23.13) | 16(17.20) |  |  | 1.448(0.748,2.804) | 0.272 |
| IgM n(%) |  |  | 0.198^b^ | 0.657 |  |  |
| low | 137(93.20) | 88(94.62) |  |  | Ref | |
| high | 10(6.80) | 5(5.38) |  |  | 1.285(0.425,3.884) | 0.657 |
| C3 n(%) |  |  | 0.914^b^ | 0.339 |  |  |
| low | 139(94.56) | 85(91.40) |  |  | Ref | |
| high | 8(5.44) | 8(8.60) |  |  | 0.612(0.221,1.690) | 0.343 |
| C4 n(%) |  |  | 0.089^b^ | 0.766 |  |  |
| low | 131(89.12) | 84(90.32) |  |  | Ref | |
| high | 16(10.88) | 9(9.68) |  |  | 1.140(0.482,2.698) | 0.766 |
| LDL n(%) |  |  | 0.063^b^ | 0.802 |  |  |
| low | 128(87.07) | 82(88.17) |  |  | Ref | |
| high | 19(12.93) | 11(11.83) |  |  | 1.107(0.501,2.445) | 0.802 |
| HDL n(%) |  |  | 1.056^b^ | 0.304 |  |  |
| low | 42(28.57) | 21(22.58) |  |  | Ref | |
| high | 105(71.43) | 72(77.42) |  |  | 0.729(0.399,1.333) | 0.305 |
| TC n(%) |  |  | 0.389^b^ | 0.533 |  |  |
| low | 129(87.76) | 79(84.95) |  |  | Ref | |
| high | 18(12.24) | 14(15.05) |  |  | 0.787(0.371,1.671) | 0.534 |
| TG n(%) |  |  | 0.757^b^ | 0.384 |  |  |
| low | 122(82.99) | 73(78.49) |  |  | Ref | |
| high | 25(17.01) | 20(21.51) |  |  | 0.748(0.388,1.441) | 0.385 |
| GLU n(%) |  |  | 0.218^b^ | 0.641 |  |  |
| low | 128(87.07) | 79(84.95) |  |  | Ref | |
| high | 19(12.93) | 14(15.05) |  |  | 0.838(0.398,1.765) | 0.641 |
| RBC n(%) |  |  | 0.162^c^ | 1.000 |  |  |
| low | 40(27.21) | 25(26.88) |  |  | Ref | |
| normal | 103(70.07) | 65(69.89) |  |  | 0.990(0.550,1.784) | 0.974 |
| high | 4(2.72) | 3(3.23) |  |  | 0.833(0.172,4.038) | 0.821 |
| HGB n(%) |  |  | 0.136^c^ | 1.000 |  |  |
| low | 62(42.18) | 39(41.94) |  |  | Ref | |
| normal | 79(53.74) | 51(54.84) |  |  | 0.974(0.572,1.661) | 0.924 |
| high | 6(4.08) | 3(3.22) |  |  | 1.258(0.297,5.324) | 0.755 |
| NE n(%) |  |  | 0.002^b^ | 0.969 |  |  |
| low | 133(90.48) | 84(90.32) |  |  | Ref | |
| high | 14(9.52) | 9(9.68) |  |  | 0.982(0.407,2.371) | 0.969 |
| MONO n(%) |  |  | 5.376^b^ | 0.020 |  |  |
| low | 79(53.74) | 64(68.82) |  |  | Ref | |
| high | 68(46.26) | 29(31.18) |  |  | 1.900(1.101,3.278) | 0.021 |
| PLT n(%) |  |  | 1.687^b^ | 0.194 |  |  |
| low | 125(85.03) | 73(78.49) |  |  | Ref | |
| high | 22(14.97) | 20(21.51) |  |  | 0.642(0.328,1.256) | 0.196 |
| LY n(%) |  |  | 4.637^c^ | 0.100 |  |  |
| low | 50(34.01) | 21(22.58) |  |  | Ref | |
| normal | 89(60.54) | 69(74.19) |  |  | 0.542(0.298,0.986) | 0.045 |
| high | 8(5.45) | 3(3.23) |  |  | 1.120(0.270,4.640) | 0.876 |
| PDW n(%) |  |  | 0.509^b^ | 0.775 |  |  |
| low | 21(14.29) | 15(16.13) |  |  | Ref | |
| normal | 115(78.23) | 73(78.49) |  |  | 1.125(0.545,2.322) | 0.750 |
| high | 11(7.48) | 5(5.38) |  |  | 1.571(0.451,5.472) | 0.478 |
| PCT n(%) |  |  | 2.096^b^ | 0.148 |  |  |
| low | 126(85.71) | 73(78.49) |  |  | Ref | |
| high | 21(14.29) | 20(21.51) |  |  | 0.608(0.309,1.197) | 0.150 |
| SII M (P25, P75) | 695.28(435.13,1473.25) | 670.57(417.90,985.73) | -1.310^a^ | 0.190 | 1.000(1.000,1.000) | 0.699 |
| NLR M (P25, P75) | 2.93 (1.84,5.62) | 2.59 (1.81,3.53) | 1.443^a^ | 0.149 | 1.009(0.953,1.069) | 0.747 |
| PLR M (P25, P75) | 161.35(123.53,212.24) | 174.17(123.30,338.62) | 1.455^a^ | 0.146 | 1.000(0.999,1.001) | 0.725 |
| SIRI M (P25, P75) | 1.42(0.83,5.32) | 1.11(0.75,2.23) | 1.936^a^ | 0.053 | 1.084(1.022,1.149) | 0.007 |
| Baseline DAS28  (P25, P75) | 5.92(4.77,6.89) | 6.07(4.85,6.73) | -0.089^a^ | 0.929 | 0.999(0.969,1.029) | 0.938 |
| TJC (P25, P75) | 12(4,20) | 12(3,20) | -0.034^a^ | 0.973 | 1.002(0.972,1.034) | 0.885 |
| SJC (P25, P75) | 6(2,16) | 6(0,14) | -0.370^a^ | 0.712 | 0.995(0.981,1.009) | 0.471 |
| VAS (P25, P75) | 55(40,70) | 55(50,65) | -0.750^a^ | 0.453 | 1.035(0.866,1.237) | 0.701 |

^a^: Z-value for Mann-Whitney U; ^b^: χ^2^-value for Chi-square test; ^c^: Fisher test; ^*^: Logistic regression; OR: ratio of ratios; CI: confidence interval.

**Supplementary Table 4. The correlation between DNA methylation levels of 12 CpG sites in the promoter region of *PON1* and prognosis of RA.**

| Position | CpG site |  | Responders (n, %) | Non-responders (n, %) | χ^2^ | *P*^a^ | OR (95%CI) | *P*^b^ |
| --- | --- | --- | --- | --- | --- | --- | --- | --- |
| Chr7:  95324546 | cg17330251­_1 | low | 43(29.25） | 17(18.28) | 3.950 | 0.139 | Ref | |
|  |  | medium | 71(48.30） | 49(52.69) |  |  | 0.573(0.293,1.119) | 0.103 |
|  |  | high | 33(22.45） | 27(29.03) |  |  | 0.487(0.228,1.039) | 0.063 |
| Chr7:  95324565 | cg17330251­_2 | low | 44(29.93） | 16(17.20) | 4.933 | 0.085 | Ref | |
|  |  | medium | 69(46.94） | 51(54.84) |  |  | 0.492(0.250,0.969) | **0.040** |
|  |  | high | 34(23.13） | 26(27.96) |  |  | 0.481(0.223,1.036) | 0.062 |
| Chr7:  95324568 | cg17330251­_3 | low | 44(29.93） | 16(17.21) | 4.968 | 0.083 | Ref | |
|  |  | medium | 68(46.26） | 52(55.91) |  |  | 0.478（0.243,0.940) | **0.032** |
|  |  | high | 35(23.81） | 25(26.88) |  |  | 0.516(0.239,1.115) | 0.093 |
| Chr7:  95324577 | cg17330251­_4 | low | 44(29.93） | 16(17.21) | 4.933 | 0.085 | Ref | |
|  |  | medium | 69(46.94） | 51(54.84) |  |  | 0.492(0.250,0.969) | **0.040** |
|  |  | high | 34(23.13） | 26(27.95) |  |  | 0.483(0.224,1.044) | 0.064 |
| Chr7:  95324582 | cg17330251­_5 | low | 36(24.49） | 24(25.81) | 3.845 | 0.146 | Ref | |
|  |  | medium | 68(46.26） | 52(55.91) |  |  | 0.857(0.455,1.615) | 0.633 |
|  |  | high | 43(29.25） | 17(18.28) |  |  | 1.679(0.779,3.621) | 0.186 |
| Chr7:  95324592 | cg17330251­_6 | low | 45(30.61） | 15(16.13) | 6.373 | **0.041** | Ref | |
|  |  | medium | 68(46.26） | 52(55.91) |  |  | 0.436(0.219,0.868) | **0.018** |
|  |  | high | 34(21.23） | 26(27.96) |  |  | 0.443(0.203,0.965) | **0.040** |
| Chr7:  95324595 | cg17330251­_7 | low | 44(29.93） | 16(17.21) | 5.214 | 0.074 | Ref | |
|  |  | medium | 67(45.58） | 53(56.99) |  |  | 0.461(0.234,0.907) | 0.025 |
|  |  | high | 36(24.49） | 24(25.80) |  |  | 0.551(0.255,1.193) | 0.131 |
| Chr7:  95324600 | cg17330251­_8 | low | 42(28.57） | 18(19.35) | 11.113 | **0.004** | Ref | |
|  |  | medium | 61(41.50） | 59(63.44) |  |  | 0.437(0.225,0.849) | **0.015** |
|  |  | high | 44(29.93） | 16(17.21) |  |  | 1.148(0.514,2.564) | 0.737 |
| Chr7:  95324616 | cg17330251­_9 | low | 44(29.93） | 16(17.21) | 4.968 | 0.083 | Ref | |
|  |  | medium | 68(46.26） | 52(55.91) |  |  | 0.478(0.243,0.941) | **0.033** |
|  |  | high | 35(23.81） | 25(26.88) |  |  | 0.513(0.238,1.108) | 0.090 |
| Chr7:  95324635 | cg17330251­_10 | low | 45(30.61） | 15(16.13) | 6.478 | **0.039** | Ref | |
|  |  | medium | 67(45.58） | 53(26.99) |  |  | 0.421(0.212,0.836) | **0.014** |
|  |  | high | 35(23.81） | 25(26.88) |  |  | 0.473(0.217,1.032) | 0.060 |
| Chr7:  95324644 | cg17330251­_11 | low | 43(29.25） | 17(18.28) | 3.669 | 0.160 | Ref | |
|  |  | medium | 69(46.94） | 51(54.84) |  |  | 0.535(0.274,1.044) | 0.067 |
|  |  | high | 35(23.81） | 25(26.88) |  |  | 0.562(0.262,1.205) | 0.138 |
| Chr7:  95324658 | cg17330251­_12 | low | 45(30.61） | 15(16.13) | 6.478 | **0.039** | Ref | |
|  |  | medium | 67(45.58） | 53(26.99) |  |  | 0.424(0.213,0.843) | **0.014** |
|  |  | high | 35(23.81） | 25(26.88) |  |  | 0.470(0.216,1.024) | 0.057 |
|  | Average | low | 43(29.25) | 17(18.28) | 3.845 | 0.146 | Ref |  |
|  |  | medium | 68(46.26) | 52(55.91) |  |  | 0.516(0.264,1.006) | 0.052 |
|  |  | high | 36(24.49) | 24(25.81) |  |  | 0.602(0.280,1.293) | 0.193 |

^a^: Chi-square test; ^b^: Logistic regression adjusted for age, sex; OR: odds ratio; CI: confidence interval;

Data are presented as *n* (%).

Bold values indicate the positive locus determined by statistical analysis.

**Supplementary Table 5. Diagnostic tests for multicollinearity of study variables.**

| Indicators | TOL | VIF |
| --- | --- | --- |
| Score | 0.992 | 1.008 |
| SIRI | 0.992 | 1.008 |

**Supplementary Table 6. Gene function of *PON1* in GeneMANIA.**

| Function | FDR | Genes in network | Genes in genome |
| --- | --- | --- | --- |
| icosanoid metabolic process | 0.00001986303635174015 | 6 | 105 |
| complement activation | 0.00003937412342129156 | 5 | 67 |
| lipoprotein particle | 0.00003937412342129156 | 4 | 23 |
| plasma lipoprotein particle | 0.00003937412342129156 | 4 | 22 |
| protein-lipid complex | 0.00003937412342129156 | 4 | 24 |
| fatty acid derivative metabolic process | 0.00004050154547433857 | 6 | 159 |
| fatty acid metabolic process | 0.000042960945139429924 | 7 | 295 |
| humoral immune response | 0.0001091908949405517 | 6 | 197 |
| hemostasis | 0.0009104755492342187 | 6 | 292 |
| coagulation | 0.0009104755492342187 | 6 | 293 |
| negative regulation of response to wounding | 0.001584223512024057 | 4 | 71 |
| regulation of plasma lipoprotein particle levels | 0.00246949409574099 | 4 | 81 |
| regulation of wound healing | 0.005517295504696719 | 4 | 101 |
| terpenoid metabolic process | 0.005540092864603084 | 4 | 103 |
| isoprenoid metabolic process | 0.008882054497498786 | 4 | 118 |
| regulation of response to wounding | 0.008901358939714063 | 4 | 120 |
| negative regulation of response to external stimulus | 0.008982249450665053 | 5 | 272 |
| negative regulation of blood coagulation | 0.008982249450665053 | 3 | 37 |
| negative regulation of hemostasis | 0.013236966119837934 | 3 | 44 |
| negative regulation of coagulation | 0.013236966119837934 | 3 | 44 |
| plasma lipoprotein particle organization | 0.015672262210462678 | 3 | 48 |
| plasma lipoprotein particle clearance | 0.015672262210462678 | 3 | 48 |
| acute inflammatory response | 0.016898068204402882 | 3 | 52 |
| negative regulation of wound healing | 0.016898068204402882 | 3 | 52 |
| protein-lipid complex subunit organization | 0.016898068204402882 | 3 | 50 |
| regulation of blood coagulation | 0.016898068204402882 | 3 | 51 |
| regulation of hemostasis | 0.01927713704951139 | 3 | 55 |
| regulation of coagulation | 0.020703618735073835 | 3 | 57 |
| regulation of humoral immune response | 0.02396360945208433 | 3 | 61 |
| blood coagulation | 0.02396360945208433 | 4 | 181 |
| response to drug | 0.024088052149469193 | 3 | 62 |
| monocarboxylic acid biosynthetic process | 0.027152419525429263 | 4 | 190 |
| organic hydroxy compound transport | 0.030316823554381644 | 4 | 197 |
| regulation of triglyceride catabolic process | 0.03665451545006137 | 2 | 12 |
| regulation of fibrinolysis | 0.03665451545006137 | 2 | 12 |
| cytolysis | 0.03665451545006137 | 2 | 12 |
| interaction with symbiont | 0.03665451545006137 | 3 | 77 |
| triglyceride catabolic process | 0.03665451545006137 | 2 | 12 |
| positive regulation of lipid localization | 0.03665451545006137 | 3 | 74 |
| cholesterol transport | 0.039590315165262145 | 3 | 81 |
| fibrinolysis | 0.039590315165262145 | 2 | 13 |
| killing by host of symbiont cells | 0.039590315165262145 | 2 | 13 |
| monooxygenase activity | 0.04464303103414669 | 3 | 85 |
| sterol transport | 0.04675570663636994 | 3 | 87 |
| arachidonic acid monooxygenase activity | 0.04825476905622549 | 2 | 15 |
| triglyceride-rich plasma lipoprotein particle | 0.04825476905622549 | 2 | 15 |
| positive regulation of coagulation | 0.05859715126687917 | 2 | 17 |
| tetrapyrrole binding | 0.05859715126687917 | 3 | 98 |
| positive regulation of hemostasis | 0.05859715126687917 | 2 | 17 |
| diterpenoid metabolic process | 0.05859715126687917 | 3 | 96 |
| lipid transport | 0.06176198735805782 | 4 | 265 |
| unsaturated fatty acid metabolic process | 0.06343157066616874 | 3 | 102 |
| positive regulation of triglyceride metabolic process | 0.06803187398426179 | 2 | 19 |
| killing of cells in other organism involved in symbiotic interaction | 0.07167215936697317 | 2 | 20 |
| fatty acid biosynthetic process | 0.07167215936697317 | 3 | 108 |
| olefinic compound metabolic process | 0.07167215936697317 | 3 | 109 |
| carboxylic ester hydrolase activity | 0.07233942227000908 | 3 | 110 |
| drug catabolic process | 0.0819515323419998 | 2 | 22 |
| carboxylic acid biosynthetic process | 0.0819515323419998 | 4 | 299 |
| blood coagulation, fibrin clot formation | 0.0819515323419998 | 2 | 22 |
| organic acid biosynthetic process | 0.0819515323419998 | 4 | 299 |
| regulation of lipid localization | 0.08326490295879228 | 3 | 120 |
| intestinal absorption | 0.08326490295879228 | 2 | 23 |
| regulation of cholesterol efflux | 0.08326490295879228 | 2 | 23 |
| lipoprotein particle receptor binding | 0.09266804954647107 | 2 | 25 |
| regulation of triglyceride metabolic process | 0.09266804954647107 | 2 | 25 |
| positive regulation of wound healing | 0.09266804954647107 | 2 | 25 |
| drug metabolic process | 0.09266804954647107 | 2 | 25 |
| plasma lipoprotein particle assembly | 0.09745945030802043 | 2 | 26 |
| positive regulation of cholesterol transport | 0.09745945030802043 | 2 | 26 |
